# Progress towards the 2020 fast track HIV/AIDS reduction targets across ages in Ethiopia as compared to neighboring countries using global burden of diseases 2017 data

**DOI:** 10.1186/s12889-021-10269-y

**Published:** 2021-02-04

**Authors:** Alemnesh H. Mirkuzie, Solomon Ali, Ebba Abate, Asnake Worku, Awoke Misganaw

**Affiliations:** 1 Ethiopian Public Health Institute (EPHI), Addis Ababa, Ethiopia; 2 University of Bergen, Bergen, Norway; 3 University of Washington, Institute for Health Metrics and Evaluation, Seattle, USA; 4 St. Paul’s Hospital Millennium Medical College, Addis Ababa, Ethiopia

**Keywords:** Eritrea, Ethiopia, East Africa, Fast track, HIV/AIDS, Kenya, HIV incidence, HIV/AIDS prevalence, HIV/AIDS related mortality, Rwanda, SDG, Tanzania, Targets, Tracking, Uganda

## Abstract

**Background:**

Sustainable Development Goal (SDG) 3.3, targets to eliminate HIV from being a public health threat by 2030. For better tracking of this target interim Fast Track milestones for 2020 and composite complementary measures have been indicated. This study measured the Fast Track progress in the epidemiology of HIV/AIDS in Ethiopia across ages compared to neighboring countries.

**Methods:**

The National Data Management Center for health’s research team at the Ethiopian Public Health Institute has analyzed the Global Burden of Disease (GBD) 2017 secondary data for the year 2010 to 2017 for Ethiopia and its neighbors. GBD 2017 data sources were census, demographic and a health survey, prevention of mother-to-child HIV transmission, antiretroviral treatment programs, sentinel surveillance, and UNAIDS reports. Age-standardized and age-specific HIV/AIDS incidence, prevalence, mortality, Disability-Adjusted Life Years (DALYs), incidence:mortality ratio and incidence:prevalence ratio were calculated with corresponding 95% confidence intervals.

**Results:**

Ethiopia and neighboring countries recorded slow progress in reducing new HIV infection since 2010. Only Uganda would achieve the 75% target by 2020. Ethiopia, Tanzania, and Uganda already achieved the 75% mortality reduction target set for 2020. The incidence: prevalence ratio for Ethiopia, Rwanda, and Uganda were < 0.03, indicating the countries were on track to end HIV by 2030. Ethiopia had an incidence: mortality ratio < 1 due to high mortality; while Kenya, Rwanda, Tanzania and Uganda had a ratio of > 1 due to high incidence. The HIV incidence rate in Ethiopia was dropped by 76% among under 5 children in 2017 compared to 2010 and the country would likely to attain the 2020 national target, but far behind achieving the target among the 15–49 age group.

**Conclusions:**

Ethiopia and neighboring countries have made remarkable progress towards achieving the 75% HIV/AIDS mortality reduction target by 2020, although they progressed poorly in reducing HIV incidence. By recording an incidence:prevalence ratio benchmark of less than 0.03, Ethiopia, Rwanda, and Uganda are well heading towards epidemic control. Nonetheless, the high HIV/AIDS mortality rate in Ethiopia for its incidence requires innovative strategies to reach out undiagnosed cases and to build institutional capacity for generating strong evidence to ensure sustainable epidemic control.

**Supplementary Information:**

The online version contains supplementary material available at 10.1186/s12889-021-10269-y.

## Background

Countries in East Africa have shown remarkable progress over the years in preventing and controlling the HIV/AIDS epidemic, yet still, they carry a high burden of HIV/AIDS in the world. Eritrea, Kenya, Rwanda, Tanzania, and Uganda close neighbors to Ethiopia have similar HIV/AIDS epidemic burden, epidemiologic patterns, mode of transmission and all are UNAIDS focus countries to end HIV/AIDS by 2030. Heterosexual encounter is still the dominant mode of HIV transmission in these countries. Although the HIV/AIDS prevalence is higher among females, there is a high disparity between males and females to access HIV testing, treatment, and care services, where males are less likely to access HIV/AIDS treatment care than females. Having such a large number of males who do not know their status, who do not take ART and not achieved virus suppression are big challenges in these countries as they could easily pass the virus to their sexual counterparts. This is highly concerning as young females in these countries are often engaged in transactional sex with these men for economic reasons. High-risk population such as female sex workers who have as high as 30% HIV prevalence have lower access to ART than the general female population in Kenya [1]. Despite 93% of the HIV positive pregnant women in the Eastern Africa region reportedly receiving ART, the rate of MTCT remains high at 10%, although it has shown a marked reduction from 18% in 2010. More than a third of HIV positive children do not have access to ART [1].

According to the 2018 UNAIDS update, the burden of HIV/AIDS in the region has shown steady decline for the past 10 years [1]. Ethiopia was one of the countries hardest hit by the HIV epidemic. Currently, the country is leading the way to HIV/AIDS epidemic control recording sustained reduction in the age standardized HIV incidence by 77% from 178 per 100,000 populations in 1990 to 40 per 100,000 populations in 2016, and the HIV/AIDS related mortality by 84% from 150 per 100,000 populations in 2005 to 24 per 100,000 populations in 2017 [2]. Country ownership of the HIV/AIDS prevention and control programs, strong political will and commitment to achieve the 90–90–90 targets by 2020 i.e. 90% of people living with HIV know their HIV status, 90% of people who know their HIV-positive status are accessing treatment and 90% of people on treatment have suppressed viral loads has been instrumental to the successes [1, 2].

Capitalizing on this sustained progress, the countries in East Africa including Ethiopia have targeted to end the AIDS epidemic from being a public health threat by 2030 endorsing Fast Track interim milestones, which targets to reduce new HIV infection and HIV/AIDS related deaths by 75% by 2020 from the 2010 baseline [1, 3, 4]. Aligned with the Fast Track target, the first Ethiopian Health Sector Transformation Plan (HSTP) set a target to reduce the adult HIV incidence by 60% and to reduce new HIV infections among children to zero by 2020 from the 2010 baseline [5, 6].

Ethiopia is one of the countries long been known for having a generalized HIV epidemic fueled by unprotected sexual intercourse similar to many East African countries. Currently, with an adult HIV prevalence of 0.9%, Ethiopia has joined the counties having a concentrated epidemic. Although, reducing HIV prevalence and incidence rates are big successes for the county, currently tracking new infection has presented a challenge as reflected on the poor progress made to achieve the first 90. This require extra efforts to identify the highest contributors of new HIV infection, groups that carry the highest-burden and infected individuals who otherwise would have been missed with the existing system. To this end, the country has considered HIV/AIDS as one of the immediately notifiable diseases and established a case-based surveillance system integrating it with index case testing. This initiative is coordinated by the Ethiopian Public Health Institute (EPHI) using the system of Public Health Emergency Management. Malawi, Kenya, and Tanzania have reported high HIV positive yield through index case testing over existing routine testing approaches [7, 8].

The main drivers of the HIV/AIDS epidemic in Ethiopia had been sexually active adults (15–49 years) and were the primary target for the national HIV/AIDS prevention and control efforts. These efforts have marked contribution to the reduced national burden of HIV/AIDS and for the recoded positive progress the country has made. However, these situations have changed, according to an urban-based HIV survey, the burden of HIV/AIDS was distributed across the age groups [9], which highlights the need for all-inclusive approaches. Lack of reliable and comprehensive data on the age-specific burden of HIV/AIDS precludes the Ethiopian Federal Ministry of Health (FMOH) and the Federal HIV/AIDS Prevention and Control Office (FHAPCO) and other concerned stakeholders from understanding the magnitude of the problem to institute targeted responses.

Outcome and impact indicators including incidence, mortality, prevalence, and Disability-Adjusted Life Years (DALYs) are commonly used epidemiological measures for tracking progress during the Millennium Development Goals era and beyond for estimating disease burden, to inform equitable resource allocation, policy formulation and for developing strategies [1, 10]. Following the calming of the HIV/AIDS epidemic globally, the UNAIDS has suggested dynamic composite measures; an incidence: mortality ratio and an incidence: prevalence ratio for better tracking of countries’ progress towards the 2030 SDG target to ending AIDS as a public health threat [1]. The objectives of this study were to track the progress on the epidemiology of HIV/AIDS towards the 2020 Fast Track milestones in Ethiopia comparing with neighboring countries in East Africa using outcome and impact indicators and the UNAIDS suggested composite measures, and to estimate age-standardized and age-specific burden of HIV/AIDS in Ethiopia since 2010.

## Methods

The Ethiopia Public Health Institute (EPHI) is a technical wing for the Ministry of Health responsible for coordination of notifiable diseases and public health emergencies. HIV is currently included under the list of notifiable health condition and established a case based surveillance system. Moreover, the institute is responsible for public health research and national health data management. The objectives of the National Data Management Center for health (NDMC) include collecting and archiving available health and health-related data; undertaking in-depth data analysis by integrating different data sources and applying robust statistical analytic methods; identify evidence gaps and research priorities and synthesize evidence for policy and decision. The center has a strong collaboration with the Institute of Health Metrics and Evaluation (IHME), the University of Washington, which produces the burden of disease estimates for 195 countries and some sub-national regions. The center also has a Burden of Disease unit and is actively involved in the estimation of national and sub-national disease burden. This study has been developed as part of the center’s activity to provide evidence for tracking Fast Track interim HIV/AIDS reduction targets using GBD 2017 data.

In this study outcome and impact indicators were measured. These include incidence which, estimated new HIV infection; prevalence, which estimated all HIV infection and mortality, which estimated all HIV/AIDS related deaths. Moreover, the study reported Years of Life Lost (YLLs), which estimated the expected number of years of life remaining when the death occurred. Here deaths at a younger age have greater weight than deaths in old age. Years Lived with Disability (YLDs), were estimated by multiplying the HIV prevalence by its disability weight. Disability-Adjusted Life Years (DALYs), which estimated the sum of years of YLLs and YLDs. The total number of DALYs in a population for 1 year could be interpreted as the distance between the current health status of the population and a hypothetical, optimal scenario where the entire population has a healthy life into old age. Incidence, Morality and DALYs rates for within-country analysis and comparing with other countries were presented in 100,000 person-year observations. Prevalence rates were presented in percentage. In the GBD analytics, age-adjusted rates have taken into consideration demographic changes in the population, such as population growth and aging. The GBD ‘first measured the change in age-standardised age-related disease burden rate (absolute and relative) from 1990 to 2017. Second, to understand the factors associated with the changes in the absolute number of age-related DALYs during this period’ … ‘constructed a decomposition analysis by expressing age-related disease burden as the product of four factors: (1) size of the adult population, (2) age structure of the adult population, (3) prevalence of age-related diseases, and (4) case fatality and disease severity of age-related diseases, which correspond to the four terms’ [11].

The study used open-access secondary data published by the GBD project for the year 2017 (S1). Due to the nature of the data used, ethical approval and consent procedures were not needed. The GBD project collects published and unpublished health data from different sources, including census, population and health registries, demographic and health surveys, and scientific publications. For countries like Ethiopia, where the population and health statistics are scares, modeling techniques are employed, taking data from other years, age groups, or similar settings to generate a complete set of estimates. The main data the GBD 2017 has incorporated for the estimation of the HIV/AIDS burden were Population and Housing Census data, Demographic and Health Surveys, and data from UNAIDS estimates. Moreover, the GBD 2017 dataset has prevalence and incidence data from antenatal care clinics and population-based sero-prevalence surveys, CD4 progression rates, HIV/AIDS related mortality with or without antiretroviral therapy (ART), and mortality from all other causes. The estimation strategy links the GBD 2017 assessment of all-cause mortality and estimation of incidence and prevalence so that for each draw from the uncertainty distribution all assumptions used in each step are internally consistent. HIV/AIDS incidence, prevalence, and death with GBD versions of the Estimation and Projection Package and Spectrum software were estimated. GBD produces point estimates and 95% Confidence Intervals (CI) for Incidence, Prevalence, Mortality, DALYs, years, countries, and age groups. One can find detailed information on the GBD methods used for estimating Global, Regional and National HIV/AIDS Incidence, Prevalence, DALYs, and Mortality published in 2016 on Lancet [12].

This study presented countries’ progress towards the 2020 interim Fast Track milestones that the United Nations General Assembly has proposed, i.e. A 75% reduction in incidence and mortality rates by 2020 from the 2010 baseline [1]. The study also used UNAIDS newly suggested complementary measures to estimate the burden of HIV/AIDS in a country and for tracking progress [1]. One of these is incidence: mortality ratio, which helps to track progress towards the SDG goal of ending AIDS as a public health threat by 2030. Combining HIV incidence and mortality measures yield a dynamic measure of the annual change in the number of people living with HIV within a given population. The ratio helps to forecast how current investments will impact future resource needs. A ratio of > 1 indicates a net increase in new HIV infections and the likely increase in the financial burden on the health system. A ratio of < 1 indicates a net reduction in prevalent HIV cases due to mortality and the likely decrease in the financial burden on the health system. However, a ratio < 1 is undesirable [1]. The other one is incidence: prevalence ratio, which indicates the average duration of time a person lives with HIV in an epidemic that remains stable over many years and helps to track progress towards the achievement of the UNAIDS objective of “Preventing HIV infections and ensuring that HIV-positive people live long and healthy lives”. For this ratio, 0.03 has been selected by the UNAIDS as an epidemic transition benchmark, which corresponds to an average life expectancy after infection of 30 years. The assumption is that with this average life expectancy, the total HIV prevalent cases will gradually fall if the number of incident cases is less than three per 100 people living with HIV [1].

## Results

### Fast track progress in Ethiopia compared to neighboring countries

#### Progress in reducing new HIV infection

**Fig. 1:**
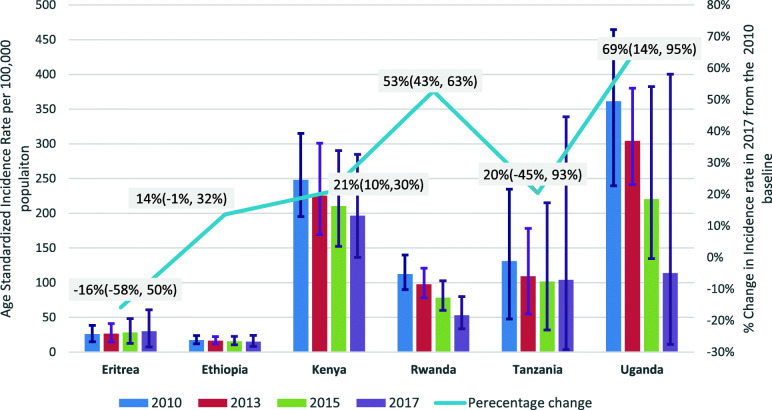
Age Standardized HIV Incidence rates per 100,000 populations from 2010 to 2017 in Ethiopian and Neighboring East African Countries. NB. The numbers are age standardized incidence rates per 100,000 populations; the numbers in bracket and the error bars indicate the 95% confidence intervals

To achieve SDG target 3.3, countries are expected to reduce new HIV infection by 75% between 2010 and 2020. The data have shown slow progress to achieve the 2020 milestone in Ethiopia and neighboring countries, but Uganda has already achieved it. Ethiopia has reduced the HIV incidence by 13.3%. In the contrary Eritrea has recorded a 13.6% increase between 2010 and 2017 (Fig. 1).

#### Progress in reducing HIV/AIDS related deaths

**Fig. 2:**
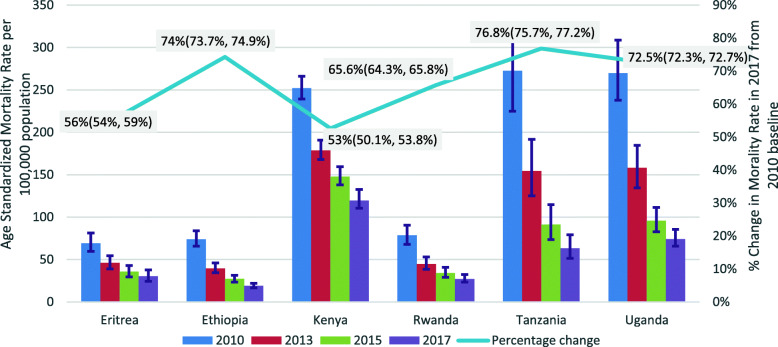
Age Standardized HIV/AIDS related Mortality rates per 100,000 populations from 2010 to 2017 in Ethiopian and Neighbouring East African Countries. NB. The numbers are age standardized HIV/AIDS related mortality rates per 100,000 populations; the numbers in bracket and the error bars indicate the 95% confidence intervals

All the countries in East Africa, neighboring Ethiopia have recorded a substantial decline in HIV/AIDS related mortality between 2010 and 2017. Ethiopia, Tanzania, Rwanda, and Uganda have already achieved the 75% mortality reduction expected to happen by 2020, while Eritrea and Kenya have achieved 56 and 53% reductions respectively by 2017 (Fig. 2).

#### Tracking countries progress using incidence: prevalence ratio

**Fig. 3:**
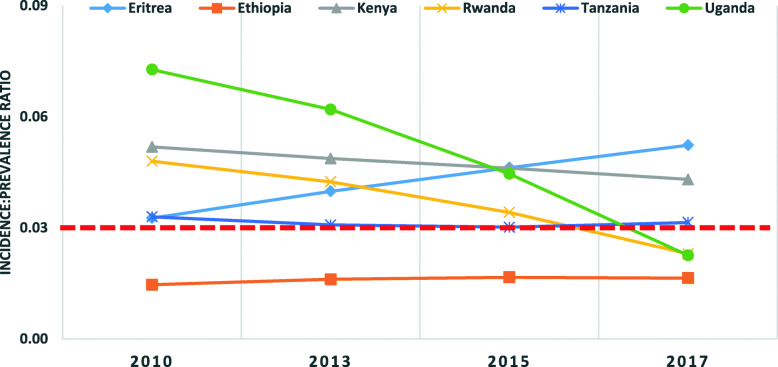
Age-standardized HIV incidence:prevalence ratio in Ethiopia and neighboring East African countries from 2010 to 2017

According to the UNAIDS, an incidence: prevalence ratio less than 0.03 indicates a country’s positive progress towards an epidemic transition as described in the methods section. The ratio for Ethiopia had been less than 0.03 since 2010, while Rwanda and Uganda achieved this benchmark in 2017. The ratio for Eritrea remained greater than 0.03 since 2010 showing a year by year increase (Fig. 3).

#### Tracking countries resource need using incidence: mortality ratio

**Fig. 4:**
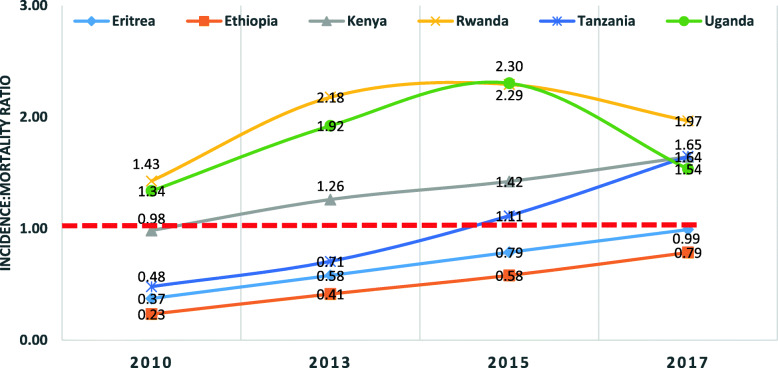
Age-standardized HIV/AIDS incidence:mortality ratio in Ethiopia and neighboring East African countries from 2010 to 2017

As shown in Fig. 4, most of the countries in East Africa, neighboring Ethiopia have more people newly infected with HIV than those dying from HIV/AIDS, which gave a ratio of greater than 1. By contrast, Ethiopia had more people dying from HIV/AIDS than those who acquired new HIV infections. In 2017, the age-standardized incidence: mortality ratio for Ethiopia was 0.79. Kenya, Rwanda, Tanzania, and Uganda had a ratio of greater than 1 due to the high rate of new infections indicating that these countries would require more resources to address the problems in the future. (Fig. 4).

### Burden of HIV/AIDS in Ethiopia across ages

#### Incidence across ages

The age-standardized annual HIV incidence rate has declined by 13% in 2017 compared to 2010 (Table 1). This corresponded to a 13% reduction in the number of people acquiring new infection from 16,676 (12,475-21,796) reported at the 2010 baseline to 14, 484 (8277-22,958) in 2017. The HIV incidence rate was highest among under 5 children from 2010 to 2015, while in 2017 the HIV incidence rate was highest among the 15–49 age group. Compared to the 2010 baseline, in 2017 the under 5 age group had recorded a 77% decline in the HIV incidence rate, while the 15–49 age group recorded a 12% increase compared to the 2010 baseline. The HIV incidence rate among 5–14 age group remained zero over the years (Table 1).

**Table 1:** All ages, age-standardized and age-specific HIV/AIDS incidence, deaths, prevalence, and DALYs rates per 100,000 populations in Ethiopia from 2010 to 2017

Age	2010	2013	2015	2017
	Rate (95% CI)	Rate (95% CI)	Rate (95% CI)	Rate (95%CI)
*Incidence*				
Under 5	51.3 (40.9, 63.8)	37.0 (29.6, 45.1)	25.1 (19.1, 32.2)	12.0 (8.1, 16.8)
5–14 years	0	0	0	0
15–49 years	21.1 (12.5, 31.6)	21.9 (14.4, 31.7)	22.8 (14.2, 34.0)	23.7 (12.4, 39.1)
50–69 years	9.4 (4.9, 15.5)	9.9 (5.6, 15.9)	10.3 (5.6, 17.0)	10.6 (4.9, 18.8)
Age-standardized	17.3 (11.9, 23.8)	16.4 (11.7, 22.4)	15.7 (10.5, 22.5)	15.0 (8.1, 24.2)
*Mortality*				
Under 5	49.4 (41.5, 58.8)	27.0 (21.5, 34.0)	18.9 (14.2, 24.5)	13.6 (10.0, 18.0)
5–14 years	17.7 (15.4, 20.1)	12.4 (10.8, 14.1)	9.4 (8.0, 10.9)	8.0 (6.6, 9.4)
15–49 years	82.4 (72.5, 92.8)	46.6 (40.2, 53.5)	32.4 (40.2, 37.9)	22.0 (18.4, 26.1)
50–69 years	87.5 (62.7, 118.2)	44.7 (30.8, 64.7)	32.2 (22.7, 45.8)	26.1 (18.0, 36.2)
Age-standardized	74.0 (65.5, 83.9)	39.6 (34.4, 46.1)	27.1 (23.4, 31.5)	19.0 (16.4, 22.1)
*DALYs*				
Under 5	4318.0 (3613.6, 5145.0)	2367.2 (1880.0, 2974.0)	1659.0 (1244.7, 2147.1)	1190.4 (875.9, 1575.4)
5–14 years	1474.4 (1296.0, 1681.7)	1037.8 (910.3, 1183.1)	789.1 (680.2, 906.6)	660.6 (552.8, 774.8)
15–49 years	4560.9 (3971.6, 5191.3)	2695.3 (2294.8, 3112.6)	1929.2 (1633.1, 2238.3)	1335.8 (1121.7, 1581.0)
50–69 years	3091.8 (2238.1, 4117.3)	1662.4 (1166.2, 2330.1)	1235.4 (898.7, 1726.3)	1028.0 (752.5, 1381.4)
Age-standardized	3905.0 (3470.0, 4352.3)	2188.9 (1915.6, 2495.7)	1533.7 (1330.7, 1776.5)	1095.6 (944.8, 1276.6)

Of the total of 14,483 (8, 277–22, 958) new HIV infections that occurred in 2017, 13.7% were among under 5 age group and 80.8% were among 15–49 age group.

#### HIV/AIDS prevalence across ages

The age-standardized HIV/AIDS prevalence rate has reduced by 23% in 2017 compared to the 2010 baseline (Table 1). This corresponded to a 15% significant reduction from 770, 657 (682, 580, 887,466) people who were reportedly living with the virus in 2010 to 657, 394 (583,399 -738,517) in 2017. Between 2010 and 2017, the highest significant HIV prevalence rate decline (64%) was observed among the under 5 age group, followed by the 5–14 age group (54%). On the contrary, the HIV prevalence rate among the 50–69 age group had shown an increase of 37% between 2010 to 2017. The HIV prevalence rate was highest among the 15–49 age group until 2010, since then the 50–69 age group has taken over the lead (Table 2).

**Table 2:** Age-standardized and age-specific HIV/AIDS prevalence in Ethiopia from 2010 to 2017

Age	Under 5% (95% CI)	5–14 years % (95% CI)	15–49 years % (95% CI)	50–69 years % (95% CI)	Age-standardized % (95% CI)
Year					
**2010**	0.25 (.21, .31)	0.49 (.40, .59)	1.34 (1.19, 1.54)	1.33 (.91, 1.98)	1.18 (1.02, 1.42)
**2013**	0.17 (.14, .20)	0.38 (.32, .46)	1.10 (1.0, 1.24)	1.46 (1.07, 2.02)	1.02 (.89, 1.19)
**2015**	0.12 (.10, .15)	0.31 (.26, .36)	0.98 (.88, 1.10)	1.61 (1.23, 2.14)	0.94 (.83, 1.08)
**2017**	0.09 (0.07, .12)	0.22 (.18, .27)	0.90 (.80, 1.02)	1.80 (1.42, 2.36)	0.91 (.80, 1.04)

Of the total 657, 394 people living with HIV/AIDS in 2017, 2.3% were under 5 years old children, 9.7% were between 5 and 14 years of age, 67.8% were between 15 and 49 years of age and 18.4% were between 50 and 69 years of age.

#### HIV/AIDS related mortality across ages

As shown in Table 1, the age-standardized mortality rate has declined by 74% from 74 deaths per 100, 000 populations in 2010 to 19 per 100,000 populations in 2017. This corresponded to a 65% significant decrease from 49, 484 (43,908-55,643) recorded deaths in 2010 due to HIV/AIDS to 17,181 (14,600-20,099) deaths in 2017. Between 2010 and 2017, the HIV/AIDS related mortality showed a 70% decline among the under 5 age group, 66.7% among the 15–49 years, 63% among the 50–69 age group and 48% decline among the 5–14 age group (Table 1).

In 2017, an estimated 17,181 people died due to HIV/AIDS, of this 13% were among < 5 age group; 13% were among 5–14 age group; 63% were among 15–49 age group and 10% were among 50–69 age group. Over the years’ mortality remained highest among the 50–69 age group. The mortality gap across the age groups has narrowed down in recent years.

#### Disability adjusted life years (DALYs) across ages

As shown in Table 2, in 2017, the age-standardized rate of DALYs was 1095.6 per 100,000 populations, which corresponded to 1,116,408 DALYs for all ages. In 2017, the age group 15–49 had the highest (1335/100,000 populations) age-specific rate of DALYs followed by the under 5 age group (1190/100,000 populations). DALYs showed a significant decline between 2010 and 2017 in all age groups, but remained highest among 15–45 age group followed by under 5 age group. Under 5 age group recorded the highest decline (72%) in the rate of DALYs between 2010 and 2017, while the age group 5–14 recorded the least (55%) (Table 1).

## Discussion

The objectives of this study were to track progress towards 2020 Fast Track target, an interim milestone for the 2030 SDG target of “ending HIV from being a public health threat” [4] in Ethiopia across ages as compared to neighboring countries. All the countries have reported remarkable progress in reducing HIV/AIDS mortality, but slow progress in reducing HIV incidence between 2010 and 2017. Ethiopia, Rwanda and Uganda have already achieved the incidence: mortality ratio of less than 0.03 and are on track to meet the SDG 2030 target with progress. The HIV/AIDS mortality rate surpassed the incidence rate in Ethiopia, contrary to most of its neighbors. This persistently high mortality would present a challenge to the government who has committed to the SDG objective of “ensuring that HIV-positive people live long and healthy lives”. The study sheds light on the burden of HIV/AIDS in Ethiopia across ages and called for tailored prevention and control interventions targeting the different age groups.

To achieve SDG target 3.3, countries are expected to reduce new HIV infection by 75% between 2010 and 2020 [1]. According to the findings, there has been slow progress to reduce HIV incidence in Ethiopia and its neighbors but Uganda. According to UNAIDS, 2018 update, although the countries in East African region were said to record remarkable progress in HIV prevention and control over the years, they lagged behind achieving the 90–90-90 targets. Rwanda has achieved the first 90 by testing over 90% of its HIV positive people, Uganda was close, while the rest of the countries were far from achieving the target. With regard to the second 90, ART coverage varied greatly, in Tanzania and Kenya it was 70–89%, in Ethiopia 50–69%, while the rest of the countries were having far less coverage [1]. Ethiopia reduced its HIV incidence only by 13.3% between 2010 and 2017 and is unlikely to achieve not only the 2020 Fast track target, but also its own HSTP plan of reducing adult HIV incidence by 60% between 2010 and 2020 [5, 6]. This is consistent with the HSTP midterm review, which highlights the broader impact poor progress has in reducing new HIV infection on the national HIV prevention and control strategies and the data gaps for monitoring progress [5, 6].

The 75% mortality reduction interim target set for 2020 is likely to be achieved by the countries. Ethiopia, Tanzania, Rwanda, and Uganda already achieved the target 3 years earlier. Eritrea and Kenya would require making accelerated progress if they are to achieve this target. Overall, the HIV/AIDS related mortality showed a declining trend across the ages in Ethiopia. The decline was remarkable among the under 5 age group followed by the 15–49 age group, whereas the age group 50–69 recorded an upward trend in recent years. Similarly, according to a study conducted in urban settings of Ethiopia, the age group 50–69 years has carried the highest burden of HIV/AIDS related mortality [9]. It is not surprising to see these results, as most of the HIV/AIDS prevention, care, and treatment services are still targeting adults (15–49 years of age) who are assumed to be the critical mass in driving the HIV/AIDS epidemic in the country, while there is little consideration that the same critical mass of the HIV/AIDS infected adults are going to age. To realize Ethiopia’s path towards epidemic control, tailored interventions are needed to improve access to HIV testing, treatment, and care services, and retention in the care and treatment program for 50 years and older adults should be considered.

In 2017, the UNAIDS has endorsed the use of incidence: prevalence ratio, a composite measure for tracking a country’s progress to end the HIV epidemic by 2030 from being a public health threat [1]. Rwanda, Uganda, and Ethiopia have already achieved, the less than 0.03 epidemic transition benchmark. This is consistent with the 2018 UNAIDS report that highlighted Ethiopia as the only country in Africa recording less than the 0.03 epidemic transition benchmark [1]. Although this finding puts Ethiopia on the lead in the HIV/AIDS epidemic control, the ever-decreasing prevalence rate in Ethiopia attributed to the high rate of mortality is worrisome and needs thorough consideration.

Incidence: mortality ratio is another composite measure the UNAIDS has endorsed to estimate the resources needed for future HIV/AIDS treatment and care services in a country. Since 2010, this ratio for Ethiopia has been less than 1 contrary to its neighbors. The country would have a reduced number of people living with HIV and may not need extra resources in the future for HIV/AIDS prevention and control [1]. However, a ratio of less than one needs cautious interpretation as suggested by the UNAIDS as it would resemble the pre-ART era if the countries have less than 81% ART coverage and a viral suppression less than 73% as in the case of Ethiopia. The reduction in HIV incidence reflects Ethiopia’s success in the fight against HIV/AIDS, while the persistently high mortality for its incidence signifies suboptimal and delayed access to HIV testing, HIV treatment care and support services in particular among the rural majority where the government should focus on to honor its commitment to the SDG objective of “ensuring that HIV-positive people live long and healthy lives”. These would require resources and renewed government commitment. The 2018 EPHIA survey reported an improved survival of HIV/AIDS positive urban residents with over 90% receiving their ART and 90% of which achieving virus suppression. Nevertheless, Ethiopia is a country with a stark urban-rural divide, not the least in population size (20% urban vs 80% rural), in HIV prevalence (3.5% urban vs 0.7% rural) and in access to health services including HIV/AIDS treatment and care [9]. At the national level about 60% of the population has never tested for HIV and over 20% of the HIV positive people do not know that they are HIV infected. About 50% of HIV positive pregnant women in Ethiopia have no access to prophylaxis ART, while access is much higher in urban than rural areas [3, 5, 13]. Nevertheless, sociocultural barriers, including poor health-seeking behavior and structural challenges to correctly map and reach out the high-risk groups before developing severe disease could have marked contributions to the persistently high mortality due to HIV/AIDS. To tackle this challenge, the government and partners working on HIV/AIDS have currently considered HIV as one of the notifiable diseases through establishing a case-based surveillance (CBS) system integrated with index case testing, and recency testing. This approach could help to increase the number of HIV cases identified to the first 90 target; to increase the positive yield which has become a resource-intensive endeavor with existing testing strategies; to improve adherence and retention to care and treatment and to ensure long term follow up as a cohort for sustainable epidemic monitoring and control.

In Ethiopia, the HIV incidence among children under 5 years of age has shown a 77% decline between 2010 and 2017. The country requires acceleration of efforts to achieve the Zero new infection target set for 2020 [5, 6]. The recorded 12% increase in HIV incidence among adults (15–49 years) in 2017 from the 2010 baseline is against the 60% reduction target the country had set for 2020. This requires revisiting current strategies and initiatives and to come up with innovative approaches to move fast forward [5, 6]. The trend data showed highest HIV incidence among the under 5 age group until 2015 compared to the other age groups. This is a reflection of the little attention given to the PMTCT program and poor HIV/AIDS treatment and care services for under 5 children in earlier years at the national and global levels [1]. In response to the UNAIDS strategic target “Zero new infection” among children, Ethiopia has introduced “Option B plus” (initiating lifelong HIV treatment for all positive mothers irrespective of immunologic status and CD4 cell count) in 2013, which has brought a remarkable progress in reducing MTCT [14–21]. However, Option B plus works only for children whose mothers have access to antenatal care and PMTCT services. In Ethiopia, access to antenatal care and PMTCT service is still low, although access is higher in urban compared to rural areas [13].

Considering the prevalence and DALYs absolute measures, Ethiopia still carries a high burden of HIV infected people, despite the country’s success story in the HIV/AIDS epidemic control. Having high prevalent cases that are not virus suppressed as in the case of the 50–69 age group, can sustain the production of new infections. According to the EPHIA data, in urban settings, the HIV prevalence is highest among the 50–64 age group (4.4%), whereby only 72.2% of the 55–64 years were virus suppressed [9]. Focusing on the declining HIV prevalence and incidence rates as a basis for financing HIV prevention and control activities could have serious consequences for a country estimated to have over 650,000 HIV infected people, whereby about 40% of them are undiagnosed.

The study used GBD 2017 data to track countries’ progress towards the 2020 fast track interim milestones. Although the GBD presents a special opportunity for countries having limited data on vital event registration, the GBD estimation has its own limitation as argued by Kelly and Wilson [22]. Ethiopia and its neighbors do not have comprehensive vital registration data, hence the GBD uses different data sources to estimate disease burden on a yearly basis. HIV/AIDS is a highly evolving condition; there might be some progress made that shape the trajectory differently where this paper did not account due to data gaps. Relying on years-old data might provide misleading information for priority setting and resource allocation. Taking some of the weaknesses of the GBD into consideration, this study compares the HIV prevalence estimates with the 2016 Ethiopian Demographic and Health Survey data and found consistency and highlights the validity of the GBD 2017 estimates for program planning and policy formulation. Moreover, the GBD 2019 and 2020 updated estimates will be available and the achievement of the targets can then be assessed.

## Conclusions

Ethiopia and neighboring countries have made remarkable progress in reducing HIV/AIDS related mortality but were, in short, of the target to reduce new HIV infections by 75% by 2020 from the 2010 baseline. Understanding the burden of HIV/AIDS along the age continuum is a step forward to sustainable epidemic control on top of instituting tailored interventions to identify new cases, to put them on treatment, and to ensure they all are virus suppressed across the ages. Current initiatives to scale up index case testing and considering HIV as a notifiable disease under the public health emergency management system show the country’s commitment and strong determination to ensure early detection of positives and retention in care and treatment to improve survival and to ensure sustainable epidemic control. These endeavors require huge financial and human resources and strengthening the surveillance, health care system and the national data systems to generate up to date and high-quality evidence for monitoring of outcome and impact and for tracking progress. Partners working on HIV need to direct its investment to support the countries to achieve national and international goals/targets through strengthening health systems and health data management systems.

## Supplementary Information

**Additional file 1.** Appendix – 2017 Global Burden of Disease HIV/AIDS estimates for tracking Fast Track progress in Ethiopia and neighboring countries in East Africa.

## Data Availability

The datasets generated during and/or analyzed for the study are available in the IHME data repository and can be accessed directly from, http://ghdx.healthdata.org/gbd-results-tool and has also been submitted as supporting file with the manuscript.

## Abbreviations

AIDS: Acquired Immuno Deficiency syndrome
ART: Antiretroviral Treatment
CBS: Case Based Surveillance
DALY: Disability Adjusted Life Years
EPHI: Ethiopian Public Health Institute
EPHIA: Ethiopian Population-Based HIV Impact Assessment
FHAPCO: Federal HIV/AIDS Prevention and Control
FMOH: Federal Ministry of Health
GBD: Global Burden of Disease
HIV: Human Immune Deficiency Virus
HSTP: Health Sector Transformation Plan
MTCT: Mother to Child HIV Transmission
NDMC: National Data Management Center for health
SDG: Sustainable Development Goal
UNAIDS: United Nations AIDS program
WHO: World Health Organization
